# Assessment of the Effectiveness of Allergic Rhinitis Medications Using a Target Trial Emulation Approach Based on Mobile Health Data

**DOI:** 10.1111/cea.70173

**Published:** 2025-11-09

**Authors:** Nuno Lourenço‐Silva, Bernardo Sousa‐Pinto, Antonio Bognanni, Matteo Martini, Michal Ordak, Giovanni Paoletti, Sara Gil‐Mata, Rita Amaral, Anna Bedbrook, Patrizia Bonadonna, Luisa Brussino, G. Walter Canonica, João Coutinho‐Almeida, Alvaro A. Cruz, Wienczyslawa Czarlewski, Mark Dykewicz, Mattia Giovannini, Bilun Gemicioglu, Juan Carlos Ivancevich, Ludger Klimek, Violeta Kvedariene, Desiree E. Larenas‐Linnemann, Manuel Marques‐Cruz, André Moreira, Marek Niedoszytko, Ana Margarida Pereira, Nikolaos G. Papadopoulos, Nhân Pham‐Thi, Frederico S. Regateiro, Sanna K. Toppila‐Salmi, Boleslaw Samolinski, Joaquin Sastre, Luís Taborda‐Barata, Tuuli Thomander, Ilgım Vardaloğlu Koyuncu, Arunas Valiulis, Leticia de las Vecillas, Maria Teresa Ventura, Jolanta Walusiak‐Skorupa, Yi‐Kui Xiang, Oliver Pfaar, João A. Fonseca, Torsten Zuberbier, Holger J. Schünemann, Danilo di Bona, Jean Bousquet, Rafael José Vieira

**Affiliations:** ^1^ MEDCIDS ‐ Department of Community Medicine, Information and Health Decision Sciences, Faculty of Medicine University of Porto Porto Portugal; ^2^ CINTESIS@RISE – Health Research Network, Faculty of Medicine University of Porto Porto Portugal; ^3^ Department of Health Research Methods, Evidence, and Impact & Department of Medicine, Evidence in Allergy Group McMaster University Hamilton Ontario Canada; ^4^ Clinical Epidemiology and Research Center (CERC), Humanitas University and Humanitas Research Hospital Milan Italy; ^5^ Department of Internal Medicine, Allergy Unit University Hospital AOU Delle Marche Ancona Italy; ^6^ Department of Clinical and Molecular Sciences Marche Polytechnic University Ancona Italy; ^7^ Department of Pharmacotherapy and Pharmaceutical Care, Faculty of Pharmacy Medical University of Warsaw Warsaw Poland; ^8^ Department of Biomedical Sciences Humanitas University, Pieve Emanuele Milan Italy; ^9^ Personalized Medicine, Asthma and Allergy IRCCS Humanitas Research Hospital Rozzano Italy; ^10^ Department of Cardiovascular and Respiratory Sciences Porto Health School, Polytechnic Institute of Porto Porto Portugal; ^11^ Department of Women's and Children's Health, Paediatric Research Uppsala University Uppsala Sweden; ^12^ Aria Montpellier France; ^13^ MASK‐Air Montpellier France; ^14^ Allergy Unit and Asthma Center Verona University Hospital Verona Italy; ^15^ Department of Medical Sciences University of Torino Torino Italy; ^16^ Allergy and Clinical Immunology Unit Mauriziano Hospital Torino Italy; ^17^ Fundacao ProAR and (Faculdade de Medicina da) Universidade Federal da Bahia Salvador Bahia Brazil; ^18^ Medical Consulting Czarlewski Levallois‐Perret France; ^19^ Section of Allergy and Immunology Saint Louis University School of Medicine Saint Louis Missouri USA; ^20^ Allergy Unit Meyer Children's Hospital IRCCS Florence Italy; ^21^ Department of Health Sciences University of Florence Florence Italy; ^22^ Department of Pulmonary Diseases Istanbul University‐Cerrahpaşa, Cerrahpaşa Faculty of Medicine Istanbul Turkey; ^23^ Institute of Pulmonology and Tuberculosis, Istanbul University‐Cerrahpaşa Istanbul Turkey; ^24^ Servicio de Alergia e Immunologia Clinica Santa Isabel Buenos Aires Argentina; ^25^ Department of Otolaryngology, Head and Neck Surgery Universitätsmedizin Mainz Mainz Germany; ^26^ Center for Rhinology and Allergology Wiesbaden Germany; ^27^ Clinic of Chest Diseases and Allergology, Faculty of Medicine Institute of Clinical Medicine, Vilnius University Vilnius Lithuania; ^28^ Department of Pathology, Faculty of Medicine Institute of Biomedical Sciences, Vilnius University Vilnius Lithuania; ^29^ Center of Excellence in Asthma and Allergy Médica Sur Clinical Foundation and Hospital México City Mexico; ^30^ EPIUnit‐Institute of Public Health University of Porto, and Laboratory for Integrative and Translational Research in Population Health (ITR) Porto Portugal; ^31^ Serviço de Imunoalergologia Centro Hospitalar Universitário São João Porto Portugal; ^32^ Basic and Clinical Immunology Unit, Department of Pathology, Faculty of Medicine University of Porto Porto Portugal; ^33^ Department of Allergology Medical University of Gdansk Gdansk Poland; ^34^ Allergy Unit, Instituto and Hospital CUF Porto Portugal; ^35^ Allergy Department, 2nd Pediatric Clinic University of Athens Athens Greece; ^36^ Ecole Polytechnique de Palaiseau Palaiseau France; ^37^ IRBA (Institut de Recherche Bio‐Médicale Des Armées), Bretigny Sur Orge France; ^38^ Université Paris Cité Paris France; ^39^ Allergy and Clinical Immunology Department Hospitais da Universidade de Coimbra, Unidade Local de Saúde de Coimbra Coimbra Portugal; ^40^ Center for Innovative Biomedicine and Biotechnology (CIBB), Faculty of Medicine, University of Coimbra Coimbra Portugal; ^41^ Faculty of Medicine Institute of Immunology, University of Coimbra Coimbra Portugal; ^42^ UBIAir ‐ Clinical & Experimental Lung Centre and CICS‐UBI Health Sciences Research Centre, University of Beira Interior Covilhã Portugal; ^43^ Department of Otorhinolaryngology University of Eastern Finland and the North Savo Wellbeing Services County Kuopio Finland; ^44^ Department of Allergy, Skin and Allergy Hospital, Inflammation Center Helsinki University Hospital and University of Helsinki Helsinki Finland; ^45^ Department of Prevention of Environmental Hazards, Allergology and Immunology Medical University of Warsaw Warsaw Poland; ^46^ Allergy Service, Fundacion Jimenez Diaz, Universidad Autonoma de Madrid, CIBERES‐ISCIII Madrid Spain; ^47^ RISE‐Health, Faculty of Health Sciences, and UBIAir‐Clinical & Experimental Lung Centre University of Beira Interior Covilhã Portugal; ^48^ Department of Immunoallergology Cova da Beira Local Health Unit Covilhã Portugal; ^49^ The Heart and Lung Center, Helsinki University Hospital, University of Helsinki Helsinki Finland; ^50^ Bagcilar Training and Research Hospital, Department of Chest Diseases Istanbul Turkey; ^51^ Clinic of Asthma, Allergy and Chronic Lung Diseases Vilnius Lithuania; ^52^ Institute of Clinical Medicine and Institute of Health Sciences, Medical Faculty of Vilnius University Vilnius Lithuania; ^53^ Department of Allergy Hospital La Paz Institute for Health Research (IdiPAZ) Madrid Spain; ^54^ University of Bari Medical School Bari Italy; ^55^ Institute of Sciences of Food Production, National Research Council (ISPA‐CNR) Bari Italy; ^56^ Department of Occupational Diseases and Environmental Health Nofer Institute of Occupational Medicine Lodz Poland; ^57^ Institute of Allergology, Charité – Universitätsmedizin Berlin, Corporate Member of Freie Universität Berlin and Humboldt‐Universität Zu Berlin Berlin Germany; ^58^ Fraunhofer Institute for Translational Medicine and Pharmacology ITMP, Immunology and Allergology Berlin Germany; ^59^ Shanghai Skin Disease Hospital, Tongji University School of Medicine Shanghai China; ^60^ Section of Rhinology and Allergy, Department of Otorhinolaryngology, Head and Neck Surgery University Hospital Marburg, Philipps‐Universität Marburg Marburg Germany; ^61^ Department of Medical and Surgical Science University of Foggia Foggia Italy

1

**Summary**

- Target trial emulation using mHealth data can support comparisons of treatment effectiveness.
- Allergic rhinitis short‐term treatment effectiveness varies according to the presence of asthma.

To the Editor,

1

Randomised controlled trials (RCTs) are the paradigm for questions on causal inference but often face challenges in generalisability due to strict eligibility criteria. In allergic rhinitis (AR), this limitation is particularly relevant, with RCTs on AR displaying an overrepresentation of patients with severe disease and not providing sufficiently detailed information on the impact of comorbidities on treatment effectiveness [1, 2, 3, 4]. Mobile health (mHealth) applications provide an opportunity to collect large‐scale patient‐reported data that can complement traditional trial evidence and broaden our understanding of treatment effectiveness in routine care [5]. However, to adequately use mHealth data for that purpose, approaches to adequately deal with confounding must be applied.

In this study, we aimed to use mHealth data to compare the short‐term effectiveness of three common AR medication classes: oral antihistamines (OAH), intranasal corticosteroids (INCS), and fixed‐combination intranasal antihistamine plus corticosteroid sprays (INAH+INCS). In particular, we aimed to compare these three medication classes on symptom relief within 24 h, while also exploring whether treatment effects differed according to the presence of self‐reported comorbid asthma. To deal with confounding, we applied a target trial emulation approach [6].

A full description of the Methods is available on https://doi.org/10.5281/zenodo.17215607. We included adult users of the MASK‐air app with self‐reported AR. MASK‐air is a validated app that allows daily reporting of AR symptoms on visual analogue scales (VAS) together with medication use. We included users who completed symptom assessments before and after taking one of the aforementioned medication classes, within a 24‐h interval. Measurements less than 60 min apart were excluded. Outcomes were changes in global, nasal, ocular, and asthma‐related symptoms (VAS, 0–100).

To address confounding, we used inverse probability of treatment weighting based on pre‐treatment symptom scores, age, sex, ARIA severity score, and asthma status. We then estimated average treatment effects using Bayesian mixed‐effects regression models, with patient and month of the year as random effects [7, 8].

A full description of the Results is available on https://doi.org/10.5281/zenodo.17215607. A total of 648 treatment days were analysed, with a median participant age of 37 years; 37.8% reported asthma. The median interval between pre‐ and post‐medication entries was 480 min (IQR = 568). Most treatment days involved OAH (64.2%), followed by INCS (25.5%) and INAH+INCS (10.3%).

Compared with OAH, both INCS and INAH+INCS were associated with significantly greater improvements in global symptoms (mean VAS difference = −4.25 [95% CrI = −6.69, −1.15] and −7.27 [−10.30, −4.07], respectively) (Table 1). Both also improved ocular symptoms, while INCS showed superiority over OAH for nasal symptoms (Table 1).

**TABLE 1:** Adjusted changes in VAS Global, Eye, Nose, and Asthma scores when comparing treatment categories.

Comparison	Difference in VAS [95% Credible Interval], probability of intervention being more effective than comparison (%)		
	All Participants	Participants with asthma	Participants without asthma
VAS global			
INCS vs. OAH	−4.25 [−6.69, −1.15], 99.3	−3.50 [−7.94, −0.39], 96.4	−5.06 [−8.27, −1.54], 99.2
INAH + INCS vs. INCS	0.62 [−2.59, 1.20], 74.1	−6.64 [−12.0, −0.17], 97.7	5.09 [−5.39, 13.3], 15.5
INAH + INCS vs. OAH	−7.27 [−10.30, −4.07], > 99.9	−3.25 [−9.53, 2.52], 88.6	−10.0 [−14.2, −6.91], > 99.9
VAS eye			
INCS vs. OAH	−4.99 [−7.22, −2.05], 99.6	−5.29 [−8.61, −0.21], 97.8	−4.32 [−8.76, −0.82], 99.0
INAH + INCS vs. INCS	−1.30 [−3.36, 1.05], 89.0	−0.25 [−7.23, 9.20], 60.0	0.01 [−8.58, 6.88], 55.6
INAH + INCS vs. OAH	−5.90 [−9.69, −2.41], 99.6	−1.74 [−7.93, 5.11], 72.8	−6.93 [−9.13, −3.75], > 99.9
VAS nose			
INCS vs. OAH	−6.37 [−8.19, −4.50], > 99.9	−6.86 [−10.0, −3.07], > 99.9	−6.69 [−9.51, −3.94], 99.9
INAH + INCS vs. INCS	−0.46 [−1.92, 1.00], 75.2	−0.10 [−10.8, 10.6], 54.5	0.05 [−11.2, 6.64], 38.7
INAH + INCS vs. OAH	0.01 [−3.40, 3.32], 51.2	10.9 [1.74, 18.5], 0.02	−9.13 [−13.3, −6.09], 99.9
VAS asthma			
INCS vs. OAH	−0.29 [−2.41, 1.04], 61.3	0.53 [−1.87, 3.99], 40.4	−0.90 [−2.06, 0.05], 97.1
INAH + INCS vs. INCS	−2.39 [−3.20, −1.56], > 99.9	−4.11 [−8.01, 2.50], 91.8	0.40 [0.26, 0.51], < 0.01
INAH + INCS vs. OAH	−5.64 [−11.9, 1.97], 94.7	−6.04 [−17.8, 7.88], 82.9	−3.13 [−4.03, 0.92], 99.8

Abbreviations: INAH + INCS, intranasal antihistamine + intranasal corticosteroids combination; INCS, intranasal corticosteroids; OAH, oral antihistamines; VAS, Visual Analogue Scale.

Among participants with asthma, INAH+INCS provided greater global symptom relief than INCS alone (mean VAS difference = −6.64, 95% CrI = −12.0, −0.17), whereas in non‐asthma participants, the combination performed worse than INCS (mean VAS difference = 5.09, 95% CrI = −5.39, 13.3).

This study demonstrates the feasibility of applying causal inference methods to mHealth data to evaluate treatment effectiveness in patients with AR. This study is, to our knowledge, the first to apply a target trial emulation approach using patient‐generated mHealth data. Such approaches allow observational datasets to emulate key components of a hypothetical RCT, thereby strengthening causal inference while leveraging the inclusiveness and ecological validity of the so‐called “real‐world” evidence.

Our findings favour intranasal therapies over OAH for symptom control. Noteworthily, we observed a differential response by asthma status. This suggests that underlying inflammatory phenotypes may modify treatment response and that asthma comorbidity should be considered when tailoring AR management. This hypothesis should, however, be confirmed by future studies.

Our findings should be interpreted considering some limitations. Firstly, there was an uneven country‐level representation, reflecting app usage patterns. However, our analysis was designed to mitigate this by focusing on individual‐level effects adjusted for key confounders. Secondly, we acknowledge the fact that the different evaluated medication classes have different pharmacodynamics. For example, the full anti‐inflammatory effect of INCS requires several days. However, (i) for most participants the assessed day was not the first in which they were doing medication, (ii) the short‐term improvements we detected are consistent with prior evidence demonstrating a clinical onset of action within 7–12 h for some molecules (of note, we detected a median 8‐h interval between first and second observations in our study) [9]. The most intriguing observation—the differential response to combination therapy (INAH+INCS) based on asthma status—is currently without a clear mechanistic explanation. We therefore present this as an exploratory, hypothesis‐generating finding that warrants further investigation, as it could be influenced by unmeasured phenotypic differences, adherence patterns, or sample size limitations within subgroups. Finally, while any study with multiple comparisons must consider the possibility of chance findings, our Bayesian framework mitigates this risk by providing probabilities of treatment superiority, offering a more nuanced interpretation than reliance on *p*‐values alone.

In summary, this study shows that mHealth data can be successfully harnessed to emulate a trial and evaluate AR treatments in real‐world conditions. Intranasal therapies (INCS and INAH+INCS) were more effective than OAH for global and ocular symptom relief. The observed effect in relation to asthma status, particularly for INAH+INCS, is a novel finding that warrants further investigation and could help refine guideline recommendations. Future studies should extend this methodology to longer follow‐up periods, larger datasets, and additional treatment strategies to continue bridging the gap between RCT and observational evidence.

## Author Contributions

N.L‐.S., B.S‐.P. and R.J.V. have participated in study design, data analysis and writing the first draft of the manuscript. A.B., D.B. and J.B. have participated in study design methodology, and writing the first draft of the manuscript. M.M., M.O., G.P. and H.J.S. have participated in methodology and critical review of the manuscript. All remaining authors have participated in data collection and critical review of the manuscript.

## Conflicts of Interest

Jean Bousquet reports personal fees from Cipla, Menarini, Mylan, Novartis, Purina, Sanofi‐Aventis, Teva, Noucor, other from KYomed‐Innov, other from Mask‐air‐SAS, outside the submitted work. Oliver Pfaar reports grants and personal fees from ALK‐Abelló, grants and personal fees from Allergopharma, grants and personal fees from Stallergenes Greer, grants and personal fees from HAL Allergy Holding B.V./HAL Allergie GmbH, grants and personal fees from Bencard Allergie GmbH/Allergy Therapeutics, grants and personal fees from Laboratorios LETI/LETI Pharma, grants and personal fees from GlaxoSmithKline, personal fees from ROXALL Medizin, personal fees from Novartis, grants and personal fees from Sanofi‐Aventis and Sanofi‐Genzyme, personal fees from Med Update Europe GmbH, personal fees from streamedup! GmbH, personal fees from Pohl‐Boskamp, grants from Inmunotek S.L., personal fees from John Wiley and Sons, AS, personal fees from Paul‐Martini‐Stiftung (PMS), personal fees from Regeneron Pharmaceuticals Inc., personal fees from RG Aerztefortbildung, personal fees from Institut für Disease Management, personal fees from Springer GmbH, grants and personal fees from AstraZeneca, personal fees from IQVIA Commercial, personal fees from Ingress Health, personal fees from Wort&Bild Verlag, personal fees from Verlag ME, personal fees from Procter&Gamble, personal fees from ALTAMIRA, personal fees from Meinhardt Congress GmbH, personal fees from Deutsche Forschungsgemeinschaft, personal fees from Thieme, grants from Deutsche AllergieLiga e.V., personal fees from AeDA, personal fees from Alfried‐Krupp Krankenhaus, personal fees from Red Maple Trials Inc., personal fees from Königlich Dänisches Generalkonsulat, personal fees from Medizinische Hochschule Hannover, personal fees from ECM Expro&Conference Management, personal fees from Technical University Dresden, grants and personal fees from Lilly, personal fees from Japanese Society of Allergy, personal fees from Forum für Medizinische Fortbildung, personal fees from Dustri‐Verlag, personal fees from Pneumolive, grants and personal fees from ASIT Biotech, personal fees from LOFARMA, personal fees from Paul‐Ehrlich‐Institut, personal fees from Almirall, outside the submitted work; and Vice President and member of EAACI Excom, member of ext. board of directors DGAKI; coordinator, main‐ or co‐author of different position papers and guidelines in rhinology, allergology and allergen‐immunotherapy; Editor‐in‐Chief (EIC) of Clinical Translational Allergy(CTA), Associate Editor (AE) of Allergy. Nhân Pham‐Thi reports personal fees from Stallergènes, personal fees from ALK, personal fees from Chiesi, personal fees from GSK, outside the submitted work. Nikolaos G. Papadopoulos reports grants from VIANEX, NUMIL HELLAS SA, VIBRANT AMERICA, personal fees from NESTLE NUTRITION INSTITUTE, GSK, HAL ALLERGY HOLDING B.V, MENARINI INTERNATIONAL OPERATIONS LUXEMBOURG SA, REGENERON PHARMACEUTICALS INC, BERLIN–CHEMIE AG. DBV TECHNOLOGIES SA, HYPROCA NUTRITION USA INC, DANONE TRADE MEDICAL B.V, MED MAPS SRL, outside the submitted work. Giovanni Paoletti reports personal fees from GSK, personal fees from LoFarma, personal fees from Sanofi, outside the submitted work. Torsten Zuberbier reports personal fees from Amgen, personal fees from AstraZeneca, personal fees from AbbVie, personal fees from ALK‐Abelló, personal fees from Almirall, personal fees from Astellas, personal fees from Bayer Health Care, personal fees from Bencard, personal fees from Berlin Chemie, personal fees from FAES Farma, personal fees from HAL Allergie GmbH, personal fees from Henkel, personal fees from Kryolan, personal fees from Leti, personal fees from L'Oreal, personal fees from Meda, personal fees from Menarini, personal fees from Merck Sharp & Dohme, personal fees from Novartis, personal fees from Nuocor, personal fees from Pfizer, personal fees from Sanofi, personal fees from Stallergenes, personal fees from Takeda, personal fees from Teva, personal fees from UCB, personal fees from Uriach, personal fees from Abivax, personal fees from Blueprint, personal fees from Celldex, personal fees from Celltrion, outside the submitted work; and Committee member, “Allergic Rhinitis and its Impact on Asthma” (ARIA), Member of the Board, German Society for Allergy and Clinical Immunology (DGAKI), Head, European Centre for Allergy Research Foundation (ECARF), President, Global Allergy and Asthma Excellence Network (GA^2^LEN), and Member, Committee on Allergy Diagnosis and Molecular Allergology, World Allergy Organisation (WAO). Luís Taborda‐Barata reports personal fees from LETI, personal fees from Sanofi, outside the submitted work. Mattia Giovannini reports personal fees from Sanofi, Thermo Fisher Scientific, outside the submitted work. The other authors declare no conflicts of interest.

## Data Availability

Data are available upon reasonable request.
